# Sero-positive HIV result disclosure to sexual partner in Ethiopia: a systematic review and meta-analysis

**DOI:** 10.1186/s12889-019-8097-y

**Published:** 2019-12-27

**Authors:** Fantahun Ayenew Mekonnen, Ayenew Molla Lakew, Kindie Fentahun Muchie, Destaw Fetene Teshome

**Affiliations:** Department of Epidemiology and Biostatistics, Institute of Public Health, College of Medicine and Health Sciences, University of Gondar, P.O.BOX: 96, Gondar, Ethiopia

**Keywords:** HIV disclosure, Factors, HIV patients, systematic review, Meta-analysis

## Abstract

**Background:**

The infection of HIV continues to be an important public health problem in Ethiopia. Disclosing own HIV positive result is crucial, and considered as a good indicator of behavior change towards HIV/AIDs. A systematic review and meta-analysis was conducted to pool the prevalence of positive HIV status disclosure to sexual partners and determine the influence of selected factors.

**Methods:**

This systematic review and meta-analysis was conducted in Ethiopia among HIV positive people receiving health care at health facilities. In this review, primary studies were searched in Medline via PubMed, Google scholar and Google up to November, 2018. Data on disclosure of HIV positive result, knowledge of partner’s HIV status and prior discussion on HIV were extracted, and effect sizes like proportion and odds ratios were pooled. Heterogeneity and publication bias were assessed by chi-square and I2, and Egger test, respectively.

**Results:**

A total of 12 studies with 4528 participants were included in to this review to estimate the prevalence of disclosure of HIV positive result to sexual partner, and 10 and 7 studies were included to determine the associations of the outcome variable with knowledge of sexual partner’s HIV status and with prior discussion on HIV, respectively. The pooled prevalence of HIV status disclosure to sexual partner was 73% (95% CI: 64, 82%). Having the knowledge of sexual partner’s HIV status [OR: 95%CI; 17.63 (7.88, 39.45)], and previous discussion on HIV [OR: 95% CI; 9.24 (5.56, 15.37)] increased the disclosure of own HIV positive result to sexual partner. The sub-group analysis indicated a prevalence of 74% in Oromia, 86% in Southern Nations Nationalities and Peoples (SNNPR), 87% in Amhara, 73% in Addis Ababa, and 54% in Tigray.

**Conclusions:**

Disclosure of HIV status to sexual partner is lower than expected. Knowledge of partner’s HIV status and previous discussion on HIV were strong predictors of HIV positive status disclosure. Strategies helpful for encouraging open HIV discussion need to be strengthened to increase HIV positive result disclosure. Furthermore, since the heterogeneity of studies is high, large nationally representative study is suggested.

## Background

HIV and AIDS still continue to be a serious global public health problem. It causes 1.8 million new infections each year. About 36.7 million people reported to live with HIV and one million people dead from HIV related illnesses in 2016. In the same year, 19.4 million people were reported to live with HIV in Eastern and Southern Africa. In these areas, 790,000 new infections reported to occur, accounting 43% of global new HIV infections, and 420,000 dead due to HIV related illnesses [1]. In Ethiopia, the problem seems to be stable though it is not as to the national and WHO target [2–4]. According to the Ethiopian Public Health Institute (EPHI) report, it was estimated that 722,248 population live with HIV, 22,827 people develop new infections and 14,872 people die of AIDS in Ethiopia [5].

Several factors are responsible for contracting the infection, which can be grouped as behavioral, socio economic and demographic factors [6–8]. Individual’s behavior is the most important factor for one’s chance of acquiring the infection [9, 10]. Early detection of HIV infection and disclosure of positive HIV test result, specifically, are necessary for sustainable control of HIV transmission [11–14].

The government of Ethiopia had been working to enable all people tested for HIV disclose their test results to their sexual partners regardless of the status of their tests [15]. Nevertheless, literature shows the prevalence of disclosure of HIV positive status is not only at its lower level but also highly variable across different parts of Ethiopia, as low as 41.8% [16] and as high as 93.1% [17]. Nevertheless, there was no an attempt to compile those evidences together to show overall status of the country in the disclosure of HIV positive result to sexual partner and the common factors contributing to it. Therefore, this systematic review and meta-analysis was conducted to summarize all available evidences of HIV positive result disclosure prevalence and key contributing behavioral characteristics. The finding will be helpful for the efforts that the country is making to develop efficient HIV prevention and control policies and strategies. Various data bases were explored to ensure if there were attempts to compile evidences of HIV status disclosure prevalence and associated factors in Ethiopia.

## Methods

Preferred Reporting Items for Systematic Reviews and Meta-Analyses (PRISMA) checklist and flow diagram were used for designing and reporting the procedure [18]. The protocol of this systematic review and meta-analysis was registered in the Prospero database: (PROSPERO 2017: CRD42017075884).

### Search strategy

A comprehensive search was carried out in Medline via PubMed, Google Scholar and Google up to November 2018. Prevalence of disclosure of HIV positive result for sexual partner and at least two associated factors were the focus of the search. The terms and/or phrases used in the search were “HIV”, “Human Immune Virus”, “STI”, “Sexually Transmitted Diseases”, “Sexually Transmitted Infection”, “Disclos*”, “expos*”, “HIV patients”, “ART users”, “Sexual partner”, “Spouse” and “Ethiopia”. There was no time limitation in the search. The search algorism was constructed using “AND” and “OR” Boolean operators.

### Eligibility criteria

We included cross sectional studies that assessed prevalence of HIV positive status disclosure to sexual partner among patients in Ethiopia, restricted to English language publications, regardless of year of publication, setting, and whether they were on ART treatment or not. Disclosure of HIV status to sexual partner was the outcome variable of this review. Knowledge of partner’s HIV status, and history of discussion on HIV related issues were the factors considered, in addition.

### Selection of studies

Two reviewers (FAM and AML) conducted literature search independently. The two reviewers were blinded to the articles’ author names, journal names and results while performing the study selection procedure. Any discrepancy between the two reviewers was resolved by negotiation or with the guidance of a third person (KFM), otherwise.

### Methodological quality assessment and data extraction

After the studies were selected, critical appraisal of the included studies was carried out using the Joanna Briggs Institute Critical Appraisal Checklist for Analytical Cross Sectional Studies [19]. The items used to appraise the selected studies were: 1) explicit inclusion criteria; 2) description of study subject and setting; 3) valid and reliable measurement of exposure; 4) objective and standard criteria used; 5) identification of confounder; 6) strategies to handle confounder; 7) outcome measurement; and 8) appropriate statistical analysis. Studies scored 50% and above of the quality scale were considered low risk. The quality assessment of the studies was carried out independently by two reviewers (FAM and AML). Any disagreement between the two reviewers was resolved by the involvement of the third reviewer (DFT). Then after, data were extracted on review variables like year of publication, study setting, study location, study design, sample size, number of HIV patients who exposed their results to their sexual partners, number of patients who reported to know their sexual partners’ HIV test results among those who exposed their HIV test results to their sexual partners and who did not expose, and the number of patients who reported to discuss on HIV with their sexual partners among patients who exposed their HIV test result to their sexual partner and who did not expose were extracted using the Joanna Briggs Institute (JBI) data extraction form for Prevalence and Incidence studies [20], and were entered in to a pre-designed Microsoft excel sheet. The reviewers (FAM, KFM and DFT) performed the data extraction.

### Statistical analysis

We examined statistical heterogeneity using the chi-square (χ^2^) test. A *p*-value less than 0.05 was considered as indicative of heterogeneity. The I^2^ with its p-value was computed, and the cut of values 25, 50 and 75%, were used to declare the degree of heterogeneity as low, moderate and high, respectively [21]. Sensitivity and sub group analyses were carried out to examine if there are primary studies and study characteristics responsible for the observed heterogeneity. The Egger test was performed to assess the publication bias [22]. For the Egger test, a *p*-value less than 0.1 was assumed as a statistically significant-evidence of publication bias present. Fixed and random effects models were considered in the meta-analysis to estimate pooled prevalence and odds ratios with 95% confidence intervals (CI) [23]. Since there was an evidence of heterogeneity, inverse variance random effects model was used as the final model of the meta-analysis. Subgroup analysis was conducted to report gender and administrative area specific effect sizes. Gender of participants (women only, and both women and men), and regions and town administrations of Ethiopia (Amhara, Oromia, SNNPR, Addis Ababa and Tigray) were considered in the sub group analysis. The Stata version 14.0 software was used for the data analysis.

## Results

### Study characteristics

**Fig. 1:**
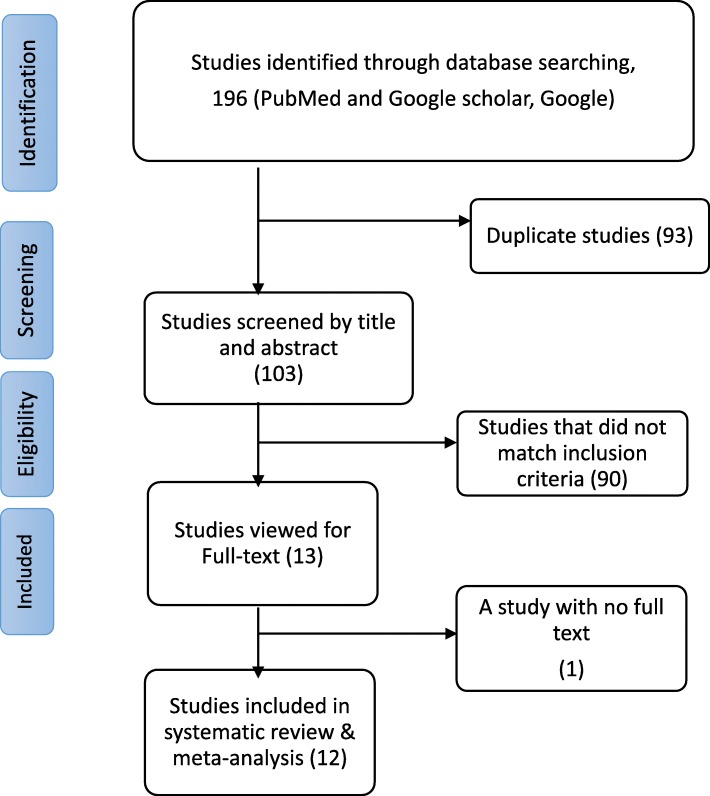
PRISMA flow diagram showing identification and inclusion of studies

Initially, a total of 196 studies were retrieved up to November, 2018 using the search strategies. Of these, 93 were duplicates and excluded. We also excluded 90 studies after screening their title and abstract. Full text was not found for one study and excluded. Finally, 12 articles with the full text were included in the meta-analysis to estimate the prevalence of HIV positive result disclosure to sexual partners [16, 17, 24–33] (Fig. 1). Out of these studies, 10 were included in the analysis to determine the association between knowledge of partner’s HIV status with disclosure of HIV status to sexual partner [16, 17, 24–26, 29–33], and 7 in the association between history of discussion on HIV with disclosure of HIV status to sexual partner [16, 17, 24, 27, 29, 31, 32]. All the 12 studies included a total of 4528 HIV patients, whereas, the 10 and 7 studies included 3815 and 2530 patients, respectively. All the included studies were conducted on four regions and one city administration of Ethiopia: four studies were from Oromia, three studies from Amhara, one study from SNNPR, one study from Addis Ababa and three studies from Tigray. Four studies included women participants only whereas 8 studies included both women and men participants. Of the 12 studies, 8 were conducted on ART patients, 2 on ART and pre ART patients and 2 on ANC attendants. Table 1 illustrates the characteristics of included studies.

**Table 1:** Characteristics and quality of included studies

Author/year	Study area	Study setting	Gender	Sample size	Prevalence of HIV status disclosure	Quality
Deribe K. et al./2008 [24]	Oromia	ART & Pre ART	Women &Men	705	90.8	Low risk
Gari T. et al./2010 [25]	SNNPR	ART	Women only	384	85.7	Low risk
Seid M. et al./2012 [17]	Amhara	ART	Women & men	360	93.1	Low risk
Erku T. et al./2012 [26]	Amhara	ART	Women & men	334	76.7	Low risk
Sendo E. et al./2013 [27]	Addis Ababa	ANC attendants	Women only	107	73.0	Low risk
Reda A. et al./2013 [28]	Oromia	ART	Women & men	606	66.3	Low risk
Alemayehu M. et al./ 2014 [29]	Tigrai	ART	Women only	315	64.0	Low risk
Alemayehu D. et al./2014 [30]	Amhara	ANC attendant	Women only	263	89.7	Low risk
Genet M. et al./2015 [31]	Tigrai	ART	Women & men	324	57.4	Low risk
Alema HB..et al./2015 [16]	Tigrai	ART & pre ART	Women & men	361	41.8	Low risk
Natae S. et al./2016 [32]	Oromia	ART	Women & men	358	84.9	Low risk
Geremew T et al./2018 [33]	Oromia	ART	Women & men	411	52.6	Low risk

### Quality assessment

The included studies were assessed for quality using the JBI critical appraisal checklist for analytical cross sectional studies, and the assessment indicated that none of the included studies were of poor in quality (Table 1).

### Prevalence of disclosure of HIV positive status for sexual partner

**Fig. 2:**
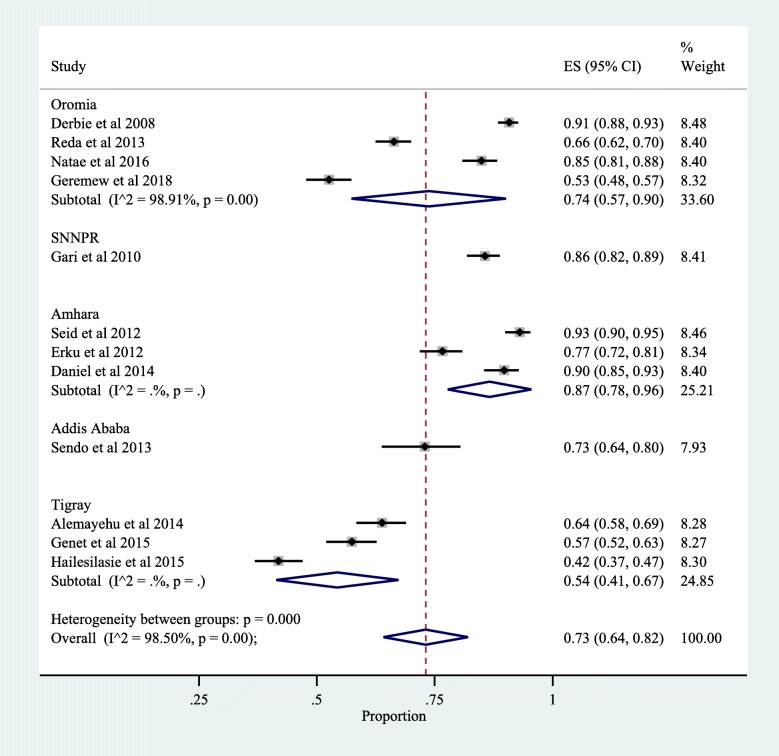
Prevalence of disclosure of HIV status to sexual partner

The overall prevalence of Disclosure of HIV positive status to sexual partner was found to be 73% (95%CI: 64, 82%), with a high level of heterogeneity (*p* < 0.001; I^2^ = 98.5%) (Fig. 2). The result of the Egger test for the prevalence of disclosure of HIV positive result for sexual partners was statistically significant (*p* < 0.05), indicating the presence of evidence of publication bias.

### Factors associated with the disclosure of HIV positive result to sexual partners

#### Knowledge of sexual partner’s HIV status

**Fig. 3:**
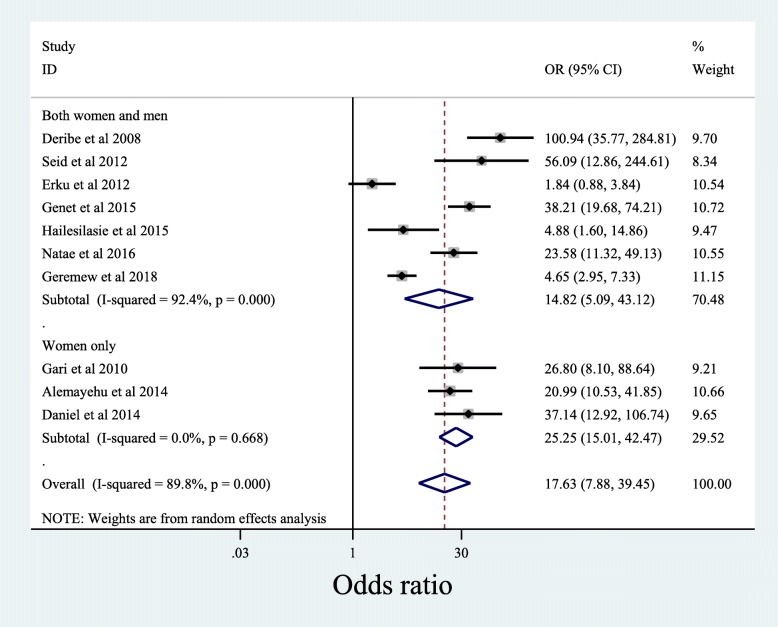
Association of knowledge of sexual partner’s HIV status with disclosure of HIV positive result

This meta- analysis shows knowledge of HIV patients about their sexual partners’ HIV status strongly associated with the patients’ disclosing of own HIV status to sexual partners. Almost the same degree and direction of association was observed at sub-population level, women only or both women and men together. Patients with the awareness of their partners’ HIV status were 17.63 times more likely to expose their positive test result to their sexual partners [OR: 95%CI; 17.63 (7.88, 39.45)], with substantial heterogeneity (*p* < 0.001; I^2^ = 89.8%). When women and men together were analyzed, significant proportion of the patients who knew their partners’ HIV status disclosed their HIV status to their partners as compared to those patients who did not know their partners’ status [OR:95%CI; 14.82 (5.09, 43.12)], with a significant level of heterogeneity (p < 0.001;I^2^ = 92.4%). When women only was considered in the analysis, significantly more women who knew their partners’ HIV status disclosed their HIV status to their sexual partner compared to those women who did not know their partners’ HIV status [OR:95%CI; 25.25 (15.01, 42.47)], with a low level of heterogeneity (*p* < 0.67;I^2^ = 0.0) (Fig. 3).

#### History of discussion on HIV

**Fig. 4:**
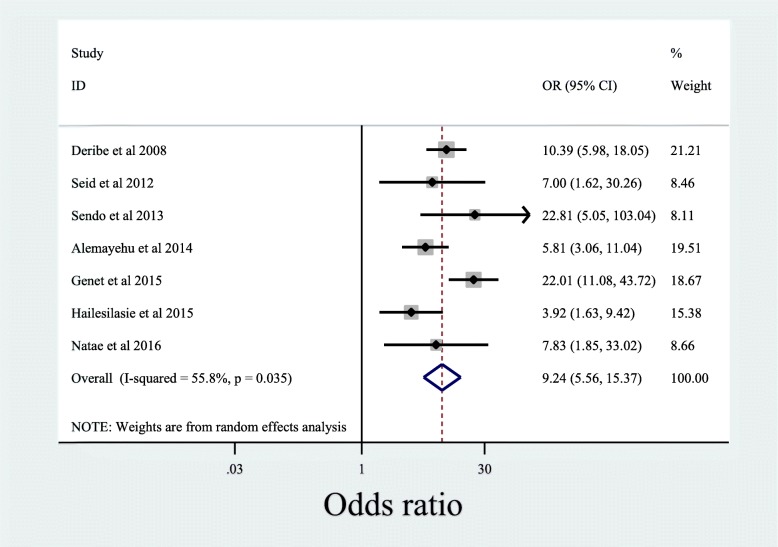
Association of history of discussion on HIV with disclosure of HIV positive result to sexual partner

This meta-analysis revealed that patients’ history of prior discussion on HIV statistically significantly associated with the disclosure of own HIV status to sexual partners. The association was almost similar in magnitude and direction at the sub-population level (women only or both women and men together). Patients with history of prior discussion on HIV were 9.24 times more likely to disclose their HIV status to their sexual partners compared to those patients who had no prior discussion on HIV [OR: 95%CI; 9.24 (5.56, 15.37)], with moderate level of heterogeneity (*p* < 0.04; I^2^ = 55.5%) (Fig. 4).

#### Subgroup analysis

We conducted subgroup analysis to see if the location of the studies (region), the type of participants (women only and both women and men together) were sources of the observed heterogeneity. The sub-group analysis indicated a prevalence of HIV status disclosure of 74% [(95%CI: 57, 90%) in Oromia region, 86% (95%CI: 82, 89%) in Southern Nations Nationalities and Peoples Region (SNNPR), 87% (95%CI: 78, 96%) in Amhara region, 73% (95%CI: 64, 80%) in Addis Ababa, and 54% (95%CI: 41, 67%) in Tigray region (Fig. 2). The heterogeneity is evident among studies conducted in Oromia region only.

## Discussion

The findings of this meta-analysis revealed that the prevalence of disclosure of HIV positive result to sexual partner was 73% (95%CI: 64, 82%). The sub-group analysis by region also showed the prevalence to be 74% (95%CI: 57, 90%) in Oromia, 86% (95%CI: 82, 89%) in Southern Nations Nationalities and Peoples Region (SNNPR), 87% (95%CI: 78, 96%) in Amhara, 73% (95%CI: 64, 80%) in Addis Ababa, and 54% (95%CI: 41, 67%) in Tigray region.

The overall HIV status disclosure prevalence in this review is similar with what was reported in Guatemala (65.6%) [34], but lower than the finding of a study in London, England (86%) [35]. In the contrary, this finding is higher than a study finding in El Salvador (59.7%) [34]. It is also higher than the results of studies conducted in South Africa that 59% of pregnant women disclosed their HIV positive results to their sexual partners [36], and in the community of rural China where 61% of participants disclosed their positive results to their sexual partners [37]. In another study conducted among African-American women to assess self-disclosure of HIV infection to significant others, 56% of them said to have reported disclosing to their sexual partners [38], which is also lower compared to the present finding. This finding is even much higher than the findings in Nicaragua (46.2%), Costa Rica (30.9%), Panama (53.0%) and Belize (28.9%) [34]*.* A number of factors can be mentioned for the difference in the prevalence of disclosure of HIV positive results to sexual partners between the finding of this review and the previous studies findings. For instance, there was evidence of difference in investment for the prevention of HIV infection between Ethiopia and Central American countries that those countries has been allocating less proportion of their economy for the prevention of the infection [39]. Other possible reasons for the differences between the current and previous studies findings might be related to the design, the scope and the study participant. To be specific, the previous studies were conducted on limited contexts like either in urban or rural; ART, Pre ART or ANC patients. For example, the study in England was conducted in the urban setting, in London only, which is believed to find a relatively higher magnitude of prevalence compared to rural community. Above all, the socio –economic status and demographic characteristics differences present between our study setting and others’ might be responsible for differences in the prevalence of disclosure of HIV positive test result.

Our study meta-analysis has also identified a strong association between own HIV status disclosure and knowledge of partner’s HIV status, and prior discussion on HIV. Study participants who knew their partners’ HIV status were more than 17.63 times more likely to disclose their HIV positive results to the sexual partners compared to those who did not have the knowledge [OR: 95%CI; 17.63 (7.88, 39.45)]. This finding coincides with a study data in London that knowledge of partner’s HIV status was the only variable significantly associated with the patients likelihood of disclosing their HIV status to their partner [35]. Coming to prior discussion on HIV, the current meta-analysis revealed that study participants who had discussion on HIV previously were 9.24 times more likely to disclose their HIV positive results to their sexual partners compared to those who had not such a previous discussion [OR: 95%CI; 9.24 (5.56, 15.37)]. This is similar with a study finding in South Africa among pregnant women that women who had discussion on HIV testing had 4 times higher chance of disclosing their positive results to sexual partners compared to those who had not have [36].

Exhibiting both of the aforementioned factors would influence behavior towards exposing own status to sexual partners, or to significant others in general, in a number of ways. The common facilitators for disclosing through which knowledge of sexual partners’ HIV status and prior discussion on HIV can work, according to several studies are; trust in the recipient of disclosure; positive experiences with previous disclosure; existence of strong social support; to gain social support; to obtain stress relief form withholding a secret; obligation and duty to inform; self-acceptance of HIV positive identity [40–42].

The authors recognized that this systematic review and meta- analysis finding might have limitations in representing the whole picture of the countries’ HIV test result disclosure prevalence since almost all of the primary studies included in the meta-analysis were not evenly distributed across the five regions of Ethiopia. Even, there were no studies conducted in some regions at all. The finding need to be interpreted cautiously as the discussion was made against primary studies due to limited previous review studies. However, there was an attempt to compare the present finding against studies considered to be large studies.

## Conclusion

Overall, the level of disclosure of HIV positive result is below what the government of Ethiopia intends to have in the country though there was much more investment to enable all patients regardless of their test results be empowered and inform their test results to their sexual partners. Therefore, the government need to strengthen the strategies helpful in advancing the behavior of the community towards open discussion on HIV testing and hence exposing own HIV test result. There need also be research aiming at determining the prevalence HIV positive test result disclosure after a specified time of knowing one’s test result, could be at within the first month after being informed the result at the latest, for instance.

## Data Availability

All data are provided in the table presented in the text.

## Abbreviations

AIDS: Acquired immune deficiency syndrome
ANC: Antenatal care
ART: Antiretroviral therapy
CI: Confidence interval
EPHI: Ethiopian Public Health Institute
HIV: Human immune virus
JBI: Joanna Briggs Institute
PRISMA: Preferred Reporting Items for Systematic Reviews and Meta-Analyses
STI: Sexually transmitted infection; WHO: World Health Organization
